# Isolation, Characterization, and Efficacy of Actinobacteria Associated with Arbuscular Mycorrhizal Spores in Promoting Plant Growth of Chili (*Capsicum flutescens* L.)

**DOI:** 10.3390/microorganisms9061274

**Published:** 2021-06-11

**Authors:** Leardwiriyakool Chaiya, Jaturong Kumla, Nakarin Suwannarach, Tanongkiat Kiatsiriroat, Saisamorn Lumyong

**Affiliations:** 1 Research Center of Microbial Diversity and Sustainable Utilization, Faculty of Science, Chiang Mai University, Chiang Mai 50200, Thailand; Leardkool@gmail.com (L.C.); jaturong_yai@hotmail.com (J.K.); suwan.462@gmail.com (N.S.); 2 Department of Biology, Faculty of Science, Chiang Mai University, Chiang Mai 50200, Thailand; 3 Department of Mechanical Engineering, Faculty of Engineering, Chiang Mai University, Chiang Mai 50200, Thailand; tanong@dome.eng.cmu.ac.th; 4 The Royal Society of Thailand, Academy of Science, Bangkok 10300, Thailand

**Keywords:** bio-inoculant, co-inoculation, plant growth promoting properties, solanaceous plants

## Abstract

Nowadays, microorganisms that display plant growth promoting properties are significantly interesting for their potential role in reducing the use of chemical fertilizers. This research study proposed the isolation of the actinobacteria associated with arbuscular mycorrhizal fungi (AMF) spores and the investigation of their plant growth promoting properties in the in vitro assay. Three actinobacterial strains were obtained and identified to the genus *Streptomyces* (GETU-1 and GIG-1) and *Amycolatopsis* (GLM-2). The results indicated that all actinobacterial strains produced indole-3-acetic acid (IAA) and were positive in terms of siderophore, endoglucanase, and ammonia productions. In the in vitro assay, all strains were grown in the presence of water activity within a range of 0.897 to 0.998, pH values within a range of 5–11, and in the presence of 2.5% NaCl for the investigation of drought, pH, and salt tolerances, respectively. Additionally, all strains were able to tolerate commercial insecticides (propargite and methomyl) and fungicides (captan) at the recommended dosages for field applications. Only, *Amycolatopsis* sp. GLM-2 showed tolerance to benomyl at the recommended dose. All the obtained actinobacteria were characterized as plant growth promoting strains by improving the growth of chili plants (*Capsicum flutescens* L.). Moreover, the co-inoculation treatment of the obtained actinobacteria and AMF (*Claroideoglomus etunicatum*) spores could significantly increase plant growth, contribute to the chlorophyll index, and enhance fruit production in chili plants. Additionally, the highest value of AMF spore production and the greatest percentage of root colonization were observed in the treatment that had been co-inoculated with *Streptomyces* sp. GETU-1.

## 1. Introduction

Chili (*Capsicum annuum* L.) is a commercially important plant that has considerable economic value as an agricultural crop. Currently, there has been an increase in demand for this plant in Thailand and throughout the world. Chili fruit contains numerous nutrients, vitamins, and minerals, and possesses a number of other health-promoting bioactive compounds that are known to be beneficial to humans [1,2]. In 2019, the Food and Agriculture Organization Statistical Database (FAOSTAT) reported that Indonesia was the largest chili producer in Southeast Asia followed by Malaysia, the Philippines, and Thailand. Importantly, the trend to increase chili production in Thailand is expected to continue to grow in the future. Over a number of years, many chemical fertilizers have been used in the cultivation of chili in order to increase the yield and quality of this economically important crop [3]. However, the excessive use of chemical fertilizers has led to environmental problems and negative impacts on human health [3,4,5]. One possible alternative to the use of chemical fertilizers involves the use of plant growth promoting microorganisms (PGPMs; actinobacteria, bacteria, filamentous fungi, and yeast) as biofertilizers that are known to be both eco-friendly and agriculturally sustainable.

Actinobacteria have been recognized as an important microorganism that can promote plant growth through the direct mechanisms (the production of growth hormones, nitrogen fixation, mineral solubilization, iron acquisition, and by increasing available plant nutrients) and through the indirect mechanisms (the inhibition of plant pathogens through the production of antimicrobial substances and cell wall degrading enzymes) [6,7]. Inoculation of plant growth promoting actinobacteria within the genera *Actinomadura*, *Actinoplanes*, *Frankia*, *Microbispora*, *Micromonospora*, *Mycobacterium*, *Nocardia*, *Nonomurea*, *Saccharopolyspora*, *Streptomyces,* and *Verrucosispor*a have been able to enhance plant growth and crop production in various plants, e.g., beans, peas, rice, tomato, and wheat [8,9,10]. Moreover, some actinobacteria have been co-inoculated with other PGPMs to effectively promote plant growth [11,12]. Numerous previous studies have focused on plant growth promoting actinobacteria that have been isolated from rhizosphere soils and are found inside various plants [10,11,12]. Currently, many researchers have focused on new types of habitats such as lichens [13], algae [14], mycorrhizosphere [15], and mangrove sediments [16] for the isolation of plant growth prompting actinobacteria, which can help overcome the challenges of discovering high efficacy strains. Thus, this study aimed to isolate actinobacteria associated with arbuscular mycorrhiza fungal (AMF) spores and investigate their plant growth promoting properties (production of IAA, siderophores, ammonia and cell wall degrading enzymes, and solubilization of phosphorus and potassium minerals). The obtained actinobacteria were identified through morphological analysis and molecular techniques. The ability of the obtained actinobacteria to tolerate conditions of drought, wide-ranging pH values, conditions of salinity, and the presence of agrochemicals was evaluated. Subsequently, the single inoculation and co-inoculation trials involving the obtained actinomyces and AMF for plant growth promotion in chili plants was conducted. The results in this study will be used to develop actinobacteria as a plant growth promotion agent that may then be used to replace chemical fertilizers and could be applied in future sustainable agricultural practices.

## 2. Materials and Methods

### 2.1. Isolation of Actinobacteria from AMF Spores

Three species of AMF (*Claroideoglomus etunicatum* PBT03, *Funneliformis mosseae* RYA08, and *Gigaspora* sp.) spores were obtained from the Research Center of Microbial Diversity and Sustainable Utilization (RCMU), Faculty of Science, Chiang Mai University, Thailand. Accordingly, *C. etunicatum* PBT03 and *F. mosseae* RYA08 were isolated from teak (*Tectona grandis* L.f.) rhizosphere soil, while *Gigaspora* sp. was isolated from the rhizosphere soil of asparagus (*Asparagus officinalis* L.) [17]. Thirty spores of each AMF species were transferred into microcentrifuge tubes and then surface sterilized following the method described by Jargeat et al. [18]. Briefly, spores were soaked three separate times in 4% chloramine T trihydrate (Merck, Darmstadt, Germany) for 10 min and the supernatant was discarded. Subsequently, AMF spores were soaked in an antibiotic aqueous solution (0.05% (*w/v*) gentamycin sulfate and 0.1% (*w/v*) streptomycin sulfate) for 30 min. Finally, AMF spores were rinsed with sterilized distilled water. The final washing water of the AMF spores was used to isolate actinobacteria on the surface of AMF spores on the actinobacteria isolate agar (Difco^TM^, New Jersey, USA) following the method described in previous studies [19,20]. The isolation plates were then incubated at 30 °C for 1 month. The individual actinobacterial colony that appeared on the isolation agar was selected and purified using the streak plate method on the International Streptomyces Project-2 medium (ISP2). Each pure actinobacterial strain was kept in 20% glycerol at −20 °C and deposited at RCMU, Chiang Mai University, Thailand.

### 2.2. Identification of Actinobacteria

#### 2.2.1. Morphological Study

The primary determination and classification of actinobacteria was evaluated according to the key criteria listed in Bergey’s Manual of Systemic Bacteriology [21]. Morphological characteristics of each actinobacterial strain were examined under a stereomicroscope (SZ-ST, Olympus, Tokyo, Japan) and light microscope (CH30, Olympus, Tokyo, Japan). Additionally, the diaminopimelic acid (DAP) types in the cell wall of each actinobacterial strain were evaluated using the thin layer chromatography (TLC) technique according to the method described by Hasegawa et al. [22].

#### 2.2.2. Molecular Study

Genomic DNA was extracted from 3-day-old samples of each actinobacterial strain cultured on ISP2 agar using a FavorPrep^TM^ Tissue Genomic DNA extraction Mini Kit (FAVORGEN^®^, Ping-Tung, Taiwan) according to the manufacturer’s instructions. The 16S ribosomal DNA (rDNA) gene was amplified using 27F (5′-AGAGTTTGATCCTG GCTCAG-3′) and 1492R (5′-GGTTACCTTGTTACGACTT-3′) primers by the polymerase chain reaction (PCR). PCR was carried out based on 20 µL of total volume per reaction and with 2 µL of genomic DNA, 1 µL of each forward and reverse primer, 10 µL of 2 × Power Taq PCR Master Mix (a mixture of i-Taq^TM^ DNA Polymerase, dNTPs, optimized buffer; iNtRON Biotechnology Inc., Gyeonggi-do, Korea), and 6 µL of sterilized double-distilled water. The amplified program was conducted by running all gene regions for 30 cycles. Initially, the denaturation procedure involved processing the specimens at 95 °C for 30 s, an annealing step at 55 °C for 30 s, an extension step at 72 °C for 60 s, and a final extension step at 72 °C for 10 min. The amplified PCR products were checked and purified using the NucleoSpin^®^ Gel and PCR Clean-up Kit (MACHEREY-NAGEL^®^, Düren, Germany).

Purified PCR products were sequenced using a commercial provider obtained from the 1st BASE Company (Kembangan, Malaysia). The sequences were assembled and edited by CodonCode, and then subjected to BLAST search in the EzBioCloud database (http://www.ezbiocloud.net/eztaxon, accessed on 12 April 2021). Multiple sequences were aligned using CLUSTAL W and then manually adjusted for phylogenetic analysis [23]. A phylogenetic tree was constructed using the neighbor-joining method [24] by applying the Kimura-2-Parameter model [25] in the MEGA software version 7.0 [26]. The confidence value of the node was supported by the bootstrap analyses based on 1000 replications.

### 2.3. Characterization of Plant Growth Promotion Properties

#### 2.3.1. Indole-3-Acetic Acid (IAA) Production

A plug (0.5 cm in diameter) of the 5-day-old culture of the actinobacterial strain grown on ISP2 agar was transferred into 18 × 180 mm test tubes that contained 5 mL of ISP2 broth. The solution of 2% L-tryptophan (Sigma-Aldrich, Beijing, China) was sterilized and added to the ISP2 broth. The culture was then inoculated and incubated in the dark at 30 °C with shaking on an orbital shaker at 150 rpm for 1 week. The supernatant was harvested by centrifugation at 11,000 rpm for 15 min. The reaction was evaluated for auxin production using the colorimetric assay by mixing the supernatant with Salkowski’s reagent (1 mL of 0.5 M FeCl_3_ in 49 mL of 35% (*w/v*) HClO_4_) [27]. A pink to red color was indicative of auxin production. The supernatant that had displayed the change in color was extracted to prepare IAA quantification with high performance liquid chromatography (HPLC) according to the method described by Kumla et al. [28]. Three replications were prepared for each actinobacterial strain.

#### 2.3.2. Siderophore Production

The potential to produce siderophores was assessed by the Chrome Azurol S (CAS) agar plate assay following the method described by Schwyn and Neilands [29]. The positive siderophore production was indicated by a change in color in the zone (yellow, orange, purple or purplish-red) around the actinobacterial colony. Three replications were made of each strain.

#### 2.3.3. Phosphate and Potassium Solubilization

The ability to solubilize the phosphorus of actinobacteria was assessed using the modified Pikoskaya (PVK) agar [30] containing tricalcium phosphate [Ca_3_(PO_4_)_2_]. In addition, modified Aleksandrov agar [31] was used to indicate whether potassium solubilization contained the potassium feldspar powder (KAlSi_3_O_8_). A plug (0.5 cm in diameter) of the 5- day-old culture of the actinobacterial strain on the ISP2 agar was inoculated onto the tested agar. After incubation at 30 °C for 2 weeks, the solubilization activity was established by the presence of a clearance zone around the colony. The solubilization efficiency (SE) was expressed in the SE index [32] by calculating the diameter of the clear zone divided by the diameter of the actinobacterial colony. Three replications were made for each actinobacterial strain.

#### 2.3.4. Cell Wall Degrading Enzyme Production

The production of the cell wall degrading enzyme was demonstrated in endoglucanase and chitinase on the agar plate assay. Endoglucanase and chitinase activities were tested on cellulose agar [33] and colloidal chitin agar [34], respectively. A plug of the actinobacterial was inoculated on the tested agar and then incubated at 30 °C for 2 weeks. The endoglucanase activity was determined by flooding the inoculated plate with 0.1% of the Congo red solution for 30 min and then rinsed with 1 M of the NaCl solution. A positive result was shown in the translucent area around the colony to indicate the hydrolysis area. In addition, a positive reaction of chitinase production resulted in the presence of a clear zone around the colony. The enzyme production was reported as an enzyme activity index (EAI) and calculated as the ratio of the halo zone diameter and colony diameter. Three replications were prepared for each strain.

#### 2.3.5. Ammonia Production

A plug of the actinobacterial strain was inoculated in 5 mL peptone water in 18 × 180 mm test tubes and shaken on a reciprocal shaker at 27 °C for 10 days. The supernatant was collected and centrifuged at 10,000 rpm for 5 min. The reaction was mixed with the supernatant along with an equal volume of Nessler’s reagent and incubated at room temperature for 3–5 min. The positive ammonia production was established by a color reaction from deep yellow to brown [35].

### 2.4. Drought, pH, and Salinity Tolerance

The tolerance to drought of the actinobacterial strain was evaluated using ISP9 agar supplemented with sorbitol powder. The amounts of sorbitol were 0, 85, 285, 405, 520, and 660 (g/L), which indicated the water activity (a_w_) at 0.998, 0.995, 0.963, 0.930, 0.912, and 0.859, respectively according to the method described by Hallsworth et al. [36]. Three replications were made for each actinobacterial strain.

Subsequently, the pH tolerance was determined in the ISP2 broth. The pH of the ISP2 broth was adjusted to 3, 4, 5, 6, 7, 8, 9, and 10 using HCl and NaOH before being autoclaved. Five milliliters of the tested broth were poured into 18 × 180 mm test tubes and inoculated with a plug of the actinobacterial strain. They were then incubated at 30 °C. The growth of actinobacteria in the tested broth was observed after 2 weeks. Three replications were prepared for each actinobacterial strain.

The salinity tolerance was determined using the ISP2 agar by adding 0, 2.5, 5, 7.5, and 10% (*w/v*) solutions of NaCl. All the tested media were adjusted to a pH of 7 with 1 N HCl or 1 N NaOH before autoclaving. One hundred microliters of 5-day-old liquid culture of each actinobacteria strain were spread onto the tested agar. Plates were incubated at 30 °C for 2 weeks and the growth of actinobacteria was observed. All replications were performed in tree replicates.

### 2.5. Agrochemical Tolerance

Tolerance to fungicides, herbicides, and insecticides was determined in the ISP2 broth following the method described by Shen et al. [37]. Tolerance was tested against five specific fungicides: Metalaxyl (Lonzan^®^), propiconazole (Conacide^®^), benomyl (Banly OD^®^), prochloraz (Jerrage Planter^®^), and captan (Orthocide 50^®^); three herbicides: Glyphosate (Glyphosate 48^®^), paraquat dichloride (Grammoxone^®^), and 2,4-d-dimethylammonium (DMA6^®^); and two insecticides: Propargite (Omite 20^®^) and methomyl (Garnet^®^). The recommended dosage for field applications of methalaxyl, propiconazole, benomyl, prochloraz, captan, glyphosate, paraquat dichloride, 2, 4-d-dimethylammonium, propargite, and methomyl were 10, 10, 7.5, 10, 15, 60, 50, 25, 15, and 10 ppm, respectively. A plug of the actinobacterial strain was inoculated in 18 × 180 mm test tubes containing 5 mL of the tested broth along with the recommended, half-recommended, and double-recommended dosages. The strain was then incubated on a shaker set at 110 rpm in the dark at 27 °C for 1 week. The cell culture was filtrated and dried at 60 °C for 48 h. The results were evaluated in terms of the tolerance index (TI) value: The dry weight of actinobacteria biomass treated with chemicals divided by the dry weight of the actinobacterial biomass that had not been treated with chemicals. Results were expressed as percentages according to the method of Fomina et al. [38]. Each treatment was performed in four replications.

### 2.6. Plant Growth Promotion in Chili by Actinobacteria and AMF Spores in Greenhouses

#### 2.6.1. Seeding and Planting Substrate

A commercially available species of Chili (*Capsicum flutescens* L.) was obtained from an agriculture store (Bird Chili, Chia Tai). Chili seeds were surface disinfected by 10% NaOCl for 5 min and rinsed three times with distilled water. The disinfected seeds were sown in trays containing sterilized soil at pH values in a range of 6.8–7.1. After 20 days, healthy seedlings of a similar size were selected for further experiments.

Sandy soil mixed with 2% (*w/w*) of the coffee waste biochar (pH value within a range of 8.19–8.33) was used as the planting substrate. The substrate was sterilized at 121 °C for 30 min in an autoclave sterilizer. After being cooled for 24 h, 50 g of the planting substrate was put into plastic pots (8 cm height × 7 cm width).

#### 2.6.2. Actinobacteria and AMF Inocula Preparation

Three obtained actinobacterial strains were used in this experiment. Each actinobacterial strain was grown on the ISP2 agar at 30 °C for 2 weeks. The spores were leached using a sterile 0.05% (*v/v*) Tween 80 aqueous solution. The suspension was adjusted to 10^6^ spores/mL using a hemocytometer.

*Claroideoglomus etunicatum* PBT03 was used as an AMF inoculum in this study. Spores were maintained at RCMU, Chiang Mai University. Spore masses were isolated from the soil inoculum according to the method described by Brundrett et al. [39]. The spore masses were then kept in Petri dishes at 4 °C until being used.

#### 2.6.3. Experimental Design

The study experiment was conducted using a complete randomized design (CRD) and eight treatments were performed (Table 1). Five replications were marked for each treatment. All treatments were repeated twice. Each treatment was controlled under natural conditions for 180 days (October 2017 to April 2018) at the Department of Biology, Faculty of Science, Chiang Mai University, Thailand (18°47′48.6″N, 98°57′33.4″E). The conditions in the greenhouse were as follows: Humidity 50–65%, temperature at 28 ± 5 °C, and maximum daily-light intensity 14,500 to 59,000 lux. Seedlings were then transplanted into plastic pots. Each plant was inoculated with one hundred AMF spores, and 20 mL of actinobacterial inoculum was added to the substrate near the root zone for each seedling. The experiment was performed with a schedule of regular watering and feeding using the modified Hoagland’s nutrient solution [40] once a week.

#### 2.6.4. Measurement of Plant Growth and Fruit Production

Values of plant height, leaf number, stem diameter, and branch number were recorded at 15, 30, 45, 60, 100, and 180 days after planting. Additionally, the chlorophyll index for the leaves of each treatment was measured at 100 and 180 days after planting using a chlorophyll meter (SPAD–502 Plus, Konica Minolta, Tokyo, Japan). The amounts of chili fruits and fruits per plant were recorded. Fruits at the mature stage were collected 180 days after the initial planting. Fruits were dried using a hot air oven at 60 °C and the dry weight was recorded as a constant weight. Whole plants in each treatment were harvested after 180 days and the dry weights of both the shoots and roots were measured.

#### 2.6.5. Measurement of AMF Root Colonization and Spore Production

At the end of the experiment (180 days), the planting substrate and plant roots for each treatment were randomly collected from each of the pots following the method described by Brundrett et al. [39]. At that point, AMF spore production and root length colonization were determined according to the method described by Brundrett et al. [39] and Phillips and Hayman [41], respectively. The percentage of AMF root colonization was calculated according to the formula of Brundrett et al. [39].

### 2.7. Statistic Analysis

Data were analyzed by the one-way analysis for variance (ANOVA) and carried out by the SPSS program version 17.0 for Windows. The significant differences (*p* ≤ 0.05) between the mean value of each treatment were considered statistically significant using Duncan’s multiple range test.

## 3. Results

### 3.1. Actinobacteria Isolation and Identification

**Figure 1:**
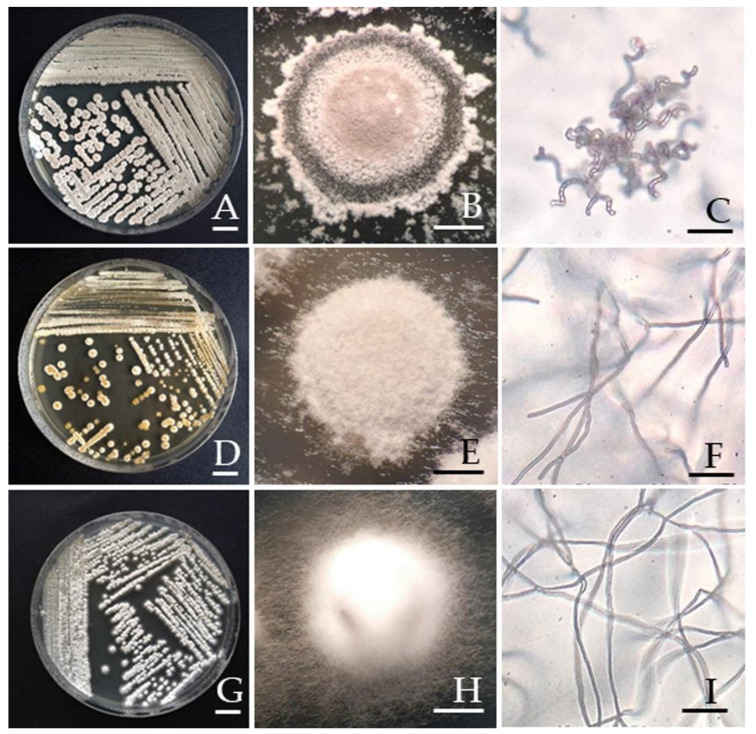
Actinobacteria isolated from AMF spores. *Streptomyces* sp. GETU-1 (**A**–**C**), *Streptomyces* sp. GIG-1 (**D**–**F**), and *Amycolatopsis* sp. GLM-2 (**G**–**I**). Cultures (**A**,**D**,**G**) and single colony (**B**,**E**,**H**) on the ISP2 agar at 30 °C for 2 weeks on the ISP2 agar. Aerial mycelium under the compound microscope (**C**,**F**,**I**). Scale bars: (**A**), (**D**), and (**G**) = 1 cm; (**B**), (**E**), and (**H**) = 2.5 mm; (**C**), (**F**), and (**I**) = 5 μm.

A total of three actinobacterial strains, GETU-1, GIG-1, and GLM-2, were isolated from the spores of *C. etunicatum* PBT03, *Gigaspora* sp., and *F. mosseae* RYA08, respectively. Colony characteristics of each actinobacterial strain are shown in Table 2 and Figure 1. The cell wall chemotype of diaminopimelic acid revealed that two strains (GETU-1 and GIG-1) presenting LL–DAP were initially classified in the streptomycete group. The remaining strain (GLM-2) was classified in the non-streptomycete group by *meso*-DAP detection (Table 1).

**Figure 2:**
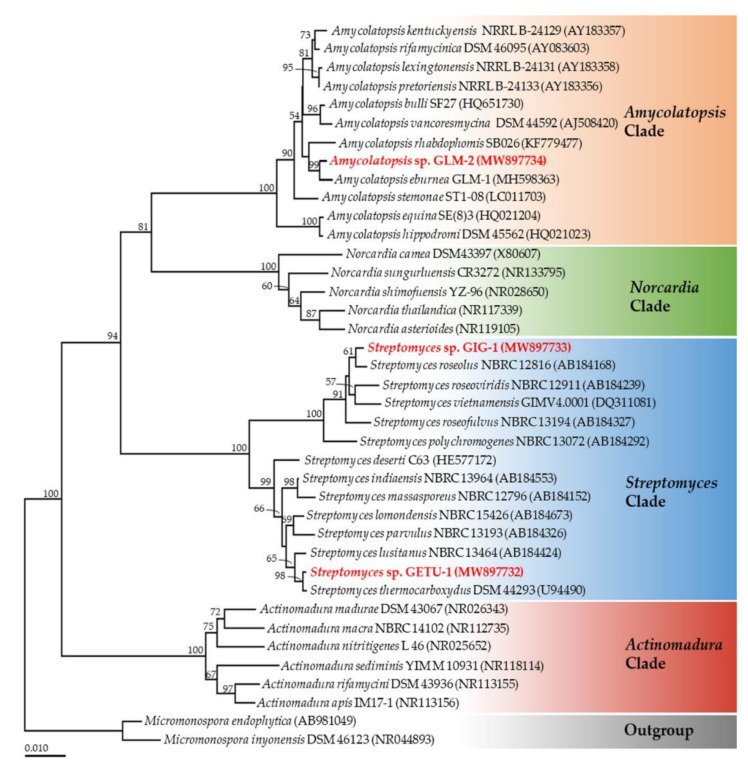
Phylogenetic tree based on 16S rRNA gene sequence with the neighbor-joining method. *Micromonospora endophytica* and *M. inyonensis* were used as outgroups. The number in each branch shows the bootstrap percentage (only values of more than 50% are shown). Bars represent 0.01 substitutions per nucleotide position. Actinobacterial strains obtained in this study are highlighted in red.

The 16S rRNA sequences of the strains GETU-1 (1343 bp), GIG-1 (1349 bp), and GLM-2 (1340 bp) were deposited in GenBank under the accession number MW897732, MW897733, and MW897734, respectively. The phylogenetic tree obtained in this study is shown in Figure 2. A phylogram was assigned into four main clades according to the genus *Actinomadura, Amycolatopsis*, *Norcadia,* and *Streptomyces*. Two sequences of *Micromonospora* were used as an outgroup. A phylogram indicated that strains GETU-1 and GIG-1 belonged to the genus *Streptomyces*. Strains GETU-1 and GIG-1 were determined to be closely related to *S. thermocarboxydus* DSM 44293 (99.65% of sequence similarity) and *Streptomyces roseolus* NBRC 12816 (99.56% of sequence similarity), respectively (Figure 2). In addition, the remaining strain, GLM-2, belonged to the genus *Amycolatopsis* and was closely related to *A. eburnea* (99.85% of sequence similarity). However, further studies would be required for absolute species identification.

### 3.2. Characterization of Plant Growth Promotion Properties

Plant growth promotion properties of all the obtained actinobacteria were characterized and results are shown in Table 3. The culture broth of all actinobacteria indicated positive auxin production by forming a red-pink color with Salkowski’s reagent. The IAA production of each strain was confirmed using the HPLC technique. It was found that IAA produced by the actinobacterial strain corresponded to the authentic IAA standard with a maximum absorption value at 279 nm and a retention time at 10.1 min according to Kumla et al. [28]. The maximum degree of IAA production (24.30 ± 4.16 µg/mL) was observed in *Streptomyces* sp. GIG-1, followed by *Streptomyces* sp. GETU-1 (4.91 ± 0.43 µg/mL) and *Amycolatopsis* sp. GLM-2 (3.04 ± 0.22 µg/mL), respectively (Table 3).

All strains were positive for siderophore production based on observations of color changes appearing in the tested agar from blue to pink around the actinobacterial colonies. All strains could not solubilize phosphate and potassium minerals. All of them were determined to be positive in terms of ammonia production. Additionally, all strains produced endoglucanase, for which the EAI value ranged from 2.15 to 3.47. However, the chitinase production was not observed in any of the strains.

All strains could be grown under conditions of water stress. *Amycolatopsis* sp. GLM-2 presented a high degree of drought tolerance in vitro and was able to be grown in agar with water availability (a_w_) at 0.897. Notably, *Streptomyces* sp. GETU-1 and *Streptomyces* sp. GIG-1 could be grown at up to a_w_ of 0.957. Furthermore, *Streptomyces* sp. GETU-1, *Streptomyces* sp. GIG-1, and *Amycolatopsis* sp. GLM-2 could be grown in agar at pH values in ranges of 5–11, 5–12, and 4–11, respectively. It was found that all strains were tolerant to salinity at up to 2.5% of NaCl.

**Figure 3:**
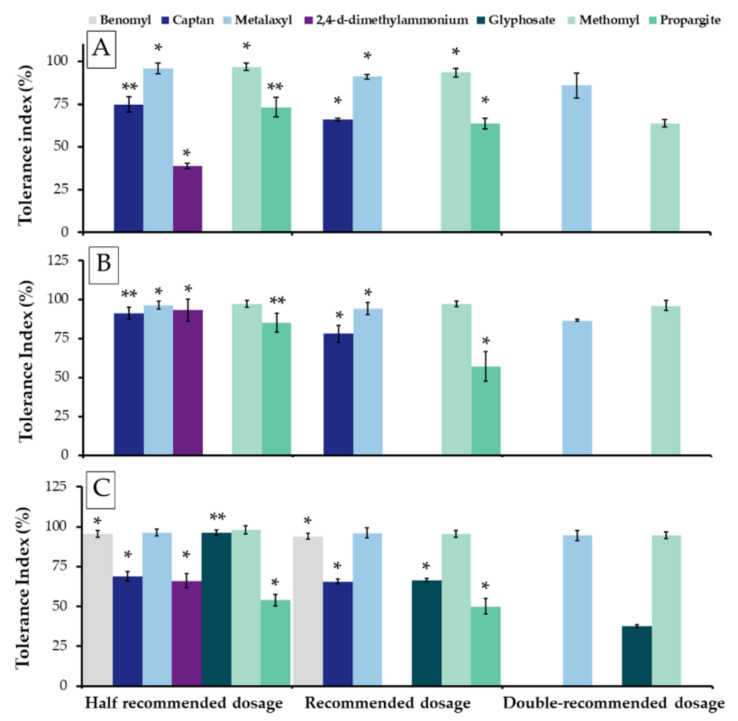
Comparison of the tolerance index of ten agrochemicals at different dosages of three actinobacteria; *Streptomyces* sp. GETU-1 (**A**); *Streptomyces* sp. GIG-1 (**B**); and *Amycolatopsis* sp. GLM-2 (**C**). Each bar indicates the standard deviation of the mean values. “*” and “**” indicates significant difference by DMRT at *p* < 0.05 in different dosages of the same agrochemical.

Agrochemical tolerance was also indicated and expressed using the tolerance index (TI). The results indicated that an increase in agrochemical concentrations reflected a decrease in the TI value of all actinobacterial strains (Figure 3). All strains could tolerate metalaxyl and methomyl in all the tested dosages (TI > 50%). Moreover, they were also found to be able to tolerate captan and propargite at half the recommended dosages. Interestingly, *Amycolatopsis* sp. GLM-2 could tolerate benomyl and glyphosate at the recommended doses. However, all strains were not tolerant of paraquat dichloride, propiconazole, and prochloraz at all the tested dosages.

### 3.3. Plant Growth Promotion in Chili by Actinobacteria and AMF Spores in Greenhouses

**Figure 4:**
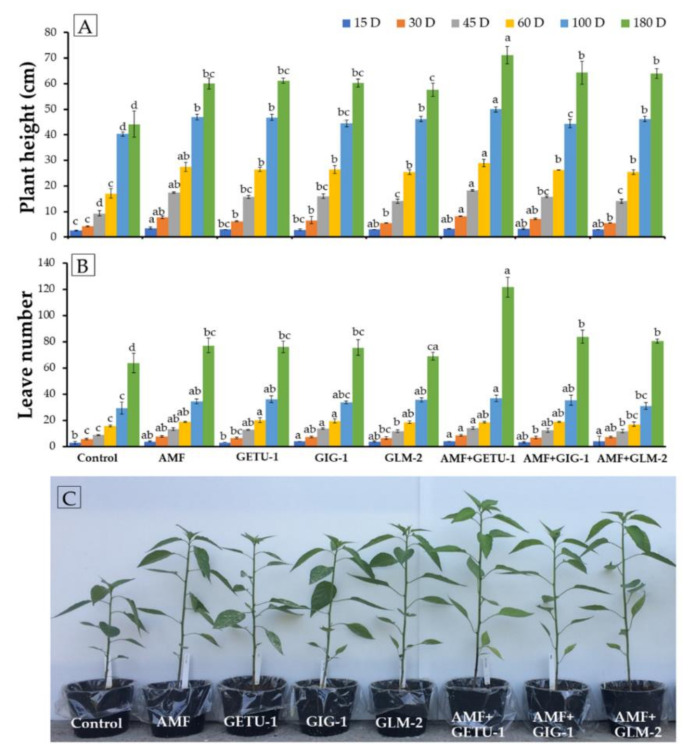
The height (**A**) and leaf number (**B**) of chili plants in each experiment during 15–180 days of planting under the greenhouse condition. The height of chili plant after 30 days of planting (**C**). The means with the standard deviation bar are shown in each graph. Different letters above each graph in the same experiment indicate that the means are significantly different by DMRT (*p* < 0.05).

The height of the chili plants and the number of leaves per plant were recorded over a period of 15–180 days after planting and results are presented in Figure 4. The results indicated that the height and number of leaves varied between the treatments. It was found that the microbial single and co-inoculation treatments could significantly increase the height and number of leaves in the chili plants. The highest values of height and leaf number were observed in the co-inoculation treatment involving AMF with *Streptomyces* sp. GETU-1 (AMF + GETU-1) after 180 days followed by co-inoculation of AMF with *Streptomyces* sp. GIG-1 and *Amycolatopsis* sp. GLM-2, respectively. However, the control treatment (uninoculated actinobacteria and AMF) produced the lowest values in terms of height and leaf numbers of the chili plants.

**Figure 5:**
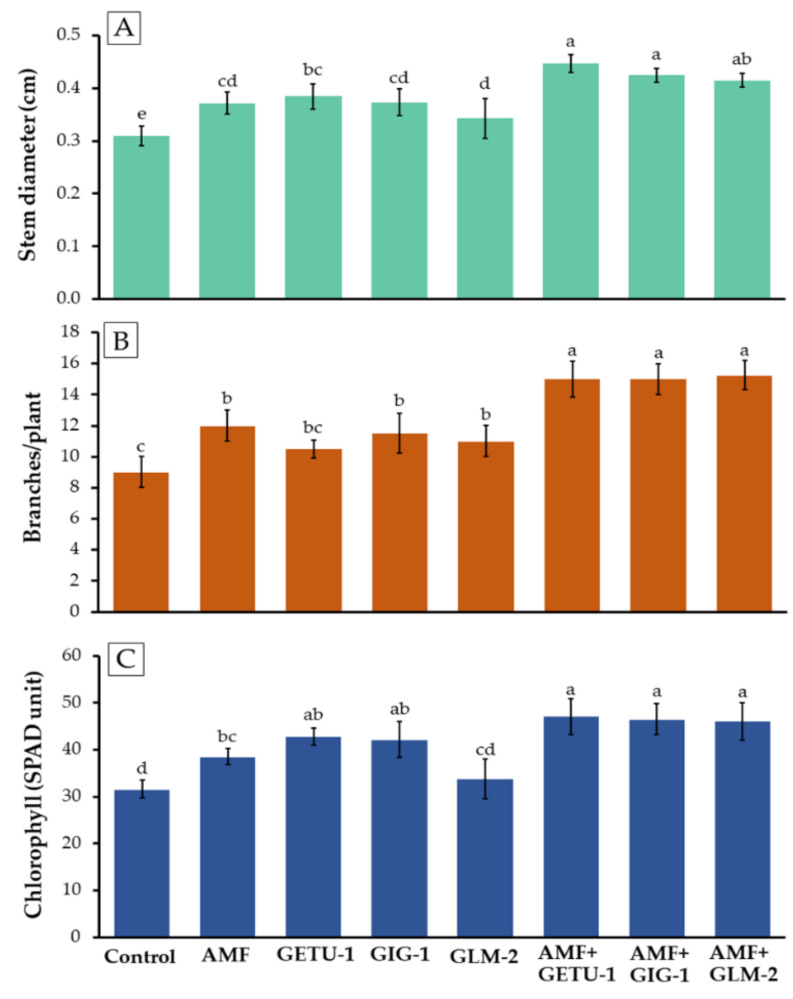
Stem diameter (**A**), branch per plant (**B**), and chlorophyll content in the leaf of chili plants (**C**) in each treatment at 180 days. The means with the standard deviation bar are shown in each graph. Different letters above each graph in the same experiment indicate that the means are significantly different by DMRT (*p* < 0.05).

The stem diameter, branch number, and chlorophyll index of the chili plants in each treatment were measured 180 days after being planted. The results indicated that the co-inoculation treatment of AMF with *Streptomyces* sp. GETU-1 (AMF + GETU-1) produced the highest stem diameter, but the values were not found to be significantly different from those of the co-inoculation treatment that employed AMF with each *Streptomyces* sp. GIG-1 (AMF + GIG-1) and *Amycolatopsis* sp. GLM-2 (AMF + GLM-2). However, the values were significantly higher than they were for the microbial single inoculation treatments and in the control (Figure 5A). Moreover, the co-inoculation treatment of AMF with each of the three actinobacterial strains could promote the branch number and chlorophyll index of the chili plants, which were significantly higher than in the other treatments (Figure 5B,C).

**Figure 6:**
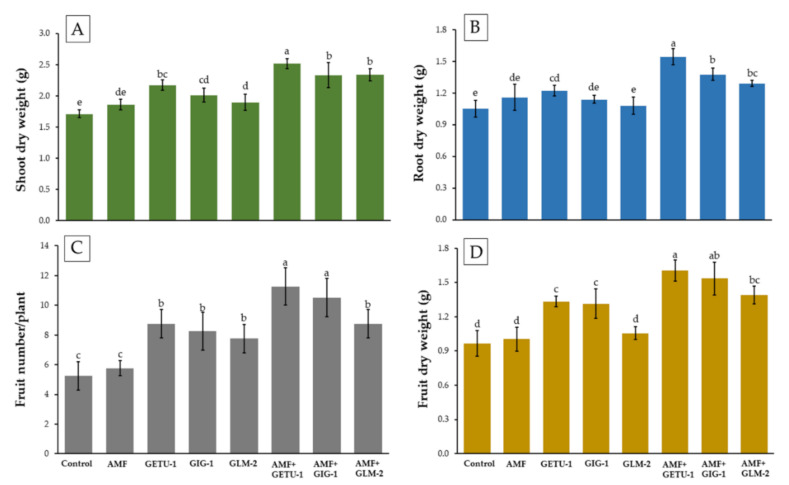
Shoot dry weight (**A**), root dry weight (**B**), fruit number per plant (**C**), and fruit dry weight per plant (**D**) of chili plants in each treatment at 180 days. The means with the standard deviation bar are shown in each graph. Different letters above each graph in the same experiment indicate that the means are significantly different by DMRT (*p* < 0.05).

The shoots and roots of chili plants were analyzed in terms of dry biomass at 180 days after being planted. Both the shoot and root biomass values were highest for the co-inoculation treatments of AMF with each actinobacterial strain were found to be significantly different from the control and single microbial inoculation treatments (Figure 6A,B).

The highest number of fruits per plant and greatest fruit dry weight were observed in the co-inoculation treatment of *Streptomyces* sp. GETU-1 and *C. etunicatum* PBT03 (AMF + GETU-1), followed by the co-inoculation of AMF and *Streptomyces* sp. GIG-1 and the co-inoculation of *Amycolatopsis* sp. GLM-2, respectively (Figure 6C,D). It was found that the values pertaining to the number of fruits per plant and the fruit dry weight in all co-inoculation treatments were significantly higher than in the single microbial inoculation and control treatments.

### 3.4. AMF Spore Density and Root Colonization

**Figure 7:**
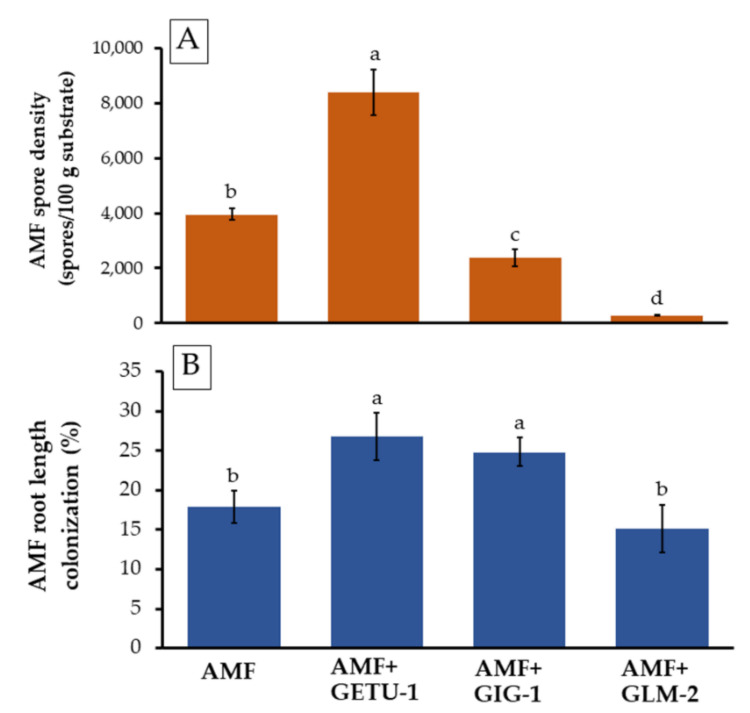
Spore density of AMF in 100 g substrate (**A**) and AMF root length colonization (**B**) in chili plants after 180 days of plantation. The means with the standard deviation bar are shown in each graph. Different letters above each graph in the same experiment indicate that the means are significantly different by DMRT (*p* < 0.05).

The AMF spore density and root colonization were observed in both single and co-inoculation experiments with actinobacteria, and the results are shown in Figure 7. This study indicated that the specimens co-inoculated with *Streptomyces* sp. GETU-1 increased both the spore production and colonization of AMF, for which the values were significantly greater than with the single AMF inoculation. In addition, the co-inoculated treatment with *Streptomyces* sp. GIG-1 only enhanced AMF root colonization. However, the co-inoculation treatment with *Amycolatopsis* sp. GLM-2 displayed a negative effect on spore production and colonization when compared with a single inoculation of AMF. It suggested that the positive result pertaining to plant growth promotion in this co-inoculation treatment was affected by *Amycolatopsis* sp. GLM-2.

## 4. Discussion

Plant growth promoting actinobacteria are typically isolated from rhizosphere soil and from inside various plants [17,18,19]. In this study, AMF spores were considered an interesting source for the isolation of plant growth promoting actinobacteria. Three actinobacterial strains, *Streptomyces* sp. GETU-1, *Streptomyces* sp. GIG-1, and *Amycolatopsis* sp. GLM-2, were obtained and the potential of these actinobacteria as PGPMs was evaluated. Several previous studies have reported that *Amycolatopsis* and *Streptomyces* have been isolated from a wide variety of habitats that are known to include AMF spores [10,11,12,19,42,43]. However, there have only been a few reports on the isolation of plant growth-promoting actinobacteria obtained from AMF spores [19,42,43]. Further studies would be required for species identification of the obtained actinobacteria in order to determine if they are representative of a potentially new species.

The properties of plant growth promoting actinobacteria are presented as various functional direct mechanisms [7,44]. In this study, three actinobacterial strains displayed properties of IAA production that involved the production of siderophores, endoglucanase, and ammonia. IAA production occurred with the presence of various microorganisms including yeast, bacteria, fungi, and actinobacteria [45,46]. In particular, actinbacteria in the genus *Streptomyces* have been reported of being capable of producing IAA [45]. Moreover, some genera *Actinomadura*, *Amycolatopsis*, *Arthrobacter*, *Nonomuraea*, *Spirillospora*, *Micromonospora*, *Nocardia,* and *Rhodococcus* have also been reported to possess this capability [47,48]. Based on the IAA production assay, all the obtained actinobacterial strains were observed to produce IAA at concentrations between 3.04 to 24.30 μg/mL. This outcome was in accordance with the IAA concentrations reported in previous research studies who found that the IAA production from actinobacteria was less than 140 μg/mL [48,49]. Moreover, previous studies have reported that the inoculation of IAA producing actinobacterial strains could effectively improve seed germination, along with the overall growth and root elongation in various plants [45,50].

Actinobacteria were able to produce siderophore for use in chelating ferric iron in limited environments that are in correlation with plant growth promotion and plant nutrient values [51,52]. Additionally, siderophores play an important role in terms of the plant protection capabilities that display activities against phytopathogens through the creation of siderophores in competitive environments [9,53]. Generally, the genera *Streptomyces* has been continuously reported to produce siderophores, which can depend upon the environment [51,54]. In this study, the *Amycolatopsis* and *Streptomyces* actinobacteria were reported to produce siderophores in accordance with the findings of a number of previous studies [54,55]. Additionally, *Actinomadura*, *Actinopolyspora*, *Micrococcus*, *Micromonospora*, *Norcardia*, *Pseaudonocardia*, *Rhodococcus*, and *Salinispora* have also been acknowledged for siderophore production [56,57]. The microbial production of ammonia plays a potentially important role in plant growth promotion via the availability of nitrogen [58]. Previous studies have found that actinobacteria, especially *Streptomyces,* produce ammonia that can lead to increased crop yields [59,60]. Interestingly, the obtained actinobacteria in this study were able to produce ammonia that supported the availability of nitrogen for plants. Actinobacteria is known to produce cell-wall degrading enzymes, especially cellulase (endoglucanase, exoglucanase, and β-glucosidase) and chitinases. Importantly, these enzymes can affect the structural integrity of the walls of the phytopathogens as targets in the biocontrol process [61,62]. However, the ability to produce enzymes was dependent upon the actinobaterial species and strain [63]. In this study, three actinobacterial strains were able to effectively produce endoglucanases. Similarly, several previous studies have reported that actinobacteria in the genera *Actinoalloteichus*, *Actinoplanes*, *Amycolatopsis*, *Arthrobacter*, *Cellulomonas*, *Kitasatospora*, *Micromonospora*, *Mycobacterium*, *Nocardia*, *Nocardioides*, *Rhodococcu*s, *Saccharomonospora*, *Streptomyces*, *Streptosporangium,* and *Thermomonospora* could produce cell-wall degrading enzymes, especially endoglucanases [61,64,65,66].

Interestingly, actinobacteria obtained in this study demonstrated tolerance to abiotic conditions of drought, pH extremes, and salinity in the in vitro assay. Accordingly, these tolerances were found to be dependent upon the actinobacterial strain. In previous studies, some species of *Amycolatopsis* (*A. arilaitensis*) and *Streptomyces* (*S. thermocarboxydus* and *S. pseudovenezuelae*) were reported to be tolerant to conditions of drought (aw 0.919–0.998) in vitro, while their benefits on plant growth and health were observed in various plants, e.g., the rice, maize, and mung beans that were grown under conditions of drought stress [43,67,68]. Our results revealed that each strain of actinobacteria displayed different levels of tolerance to pH and salinity levels and that they could be grown under both acidic and alkaline (pH 4–11) conditions. Thus, they could be grown and survive under general soil conditions (pH 6–8) and in soil that is extremely acidic or alkaline. Notably, Poomthongdee et al. [69] found the actinobacterial genera *Allokutzneria, Amycolatopsis*, *Mycobacterium, Nocardia, Nonomuraea, Saccaropolyspora, Streptaeidiphilus, Streptomyces,* and *Verrucosispora* could survive in the extremely acidic soil of rice and rubber tree plants. Sreevidya et al. [70] found that four *Streptomyces* sp. isolates can survive in extremely alkaline soil and promote the growth of the chick pea (*Cicer arietinum* L.) under field conditions. In the present investigation, actinobacteria were successfully grown with the highest NaCl concentration recorded at 2.5%. The salinity tolerance of actinobacteria depended upon the species and strain of the actinobacteria, as well as the NaCl concentration. For example, Tresner et al. [71] found that 1300 *Streptomyces* strains exhibited different degrees of salinity tolerance (at 4% up to 13% NaCl), which varied according to species and strain. *Streptomyces* sp. PGPA39 [72] and *S. rochei* SM [70] were tolerant of NaCl concentrations at up to 6 and 20%, respectively. Generally, previous studies reported that the drought, salinity, and pH levels of soil are important factors for microbial grow and survival [70,71,72,73]. Therefore, tolerance to drought, salinity, and pH should be the criteria for selection for microbial utilization. However, the actinobacteria obtained in this study for the promotion of plant growth under conditions of drought, pH extremes, and salinity, in both greenhouse and the field experiments, should be further investigated in future studies.

Our results indicate that each actinobacteria strain was tolerant of agrochemicals (fungicides, herbicides, and pesticides) at different levels depending upon the specific strain. All strains were able to tolerate commercial insecticides (propargite and methomyl) and fungicide (captan) at the recommended dosages for field application. However, an increase in the concentration of agrochemicals could reduce the growth and TI values of each strain. Our results are in accordance with other previously published findings that determined that the genera *Achromobacter*, *Arthrobacter,* and *Streptomyces* could be tolerant of many agrochemicals [70,74,75]. However, their tolerance ability was reduced when the concentration of agrochemicals was increased [70,74]. Sreevidya et al. [70] found that four *Streptomyces* sp. isolates were tolerant to certain fungicides including bavistin, thiram, and captan, but were susceptible to benlate and ridomil at field application levels. In addition, *Achromobacter* sp. ANB-1 and *Arthrobacter globiformis* D47 could be tolerant to phenylurea herbicides at the recommended field dosages [75,76]. Therefore, information on the agrochemical tolerance of the obtained actinobacteria in this study would be beneficial in field applications as the crops could be treated with this actinobacteria in combination with insecticides and fungicides under the recommend dosages.

This study found that the single inoculation of each of the three actinobacterial strains and AMF could enhance the growth and yield production of chili plants. Thus, the three actinobacterial strains in this study can be characterized as plant growth promoting actinobacteria. Our ultimate determination is in agreement with the findings of other previously published reports which found that the inoculation of PGPMs, including actinobacteria and AMF, could promote the growth and development of various plants [8,9,10]. Furthermore, previous studies have reported that co-inoculation with PGPMs could be more effective in terms of promoting plant growth and yield production than single strain inoculation of PGPM [77,78,79,80]. In the present study, co-inoculation of actinobacteria along with AMF spores resulted in an increase in the plant growth promotion of chili plants and yield production when compared with the single inoculation of each microorganism and the non-inoculated treatments. Franco-Correa et al. [77] and El-Sayed et al. [79] reported that the co-inoculation of the selected strains of *Streptomyces* and *Thermobifida* with AMF spores of *Glomus mosseae* increased the growth of white clover plants (*Trifolium repens*) and faba beans (*Vicia faba*) to a higher degree than with the single inoculation of each microorganism and in the non-inoculation treatments. The co-inoculation of *Frankia actinomycestes* with AMF (*G. aggregatum*) significantly improved nitrogen fixation and the growth of seedlings of sea-buckthorn (*Hippophae tibetana*) [78]. Anfrade et al. [80] found that the co-inoculation of *Frankia* and AMF (*G. clarum* and *G. margarita*) on river oak trees (*Casuarina cunninghamiana*) improved the survival and growth of seedlings in degraded and low fertility soil. However, these results were significantly different from those of the *Frankia* and AMF control plants. Moreover, yields of shoot length and root dry weight of corn (*Zea mays*) seedlings under conditions of co-inoculation that involved *Streptomyces* sp. W77 and W43N with AMF (*Rhizophagus irregularis*) were higher than for corn seedlings that had been received from a single inoculation of each microorganism.

In this study, the obtained actinobacteria *Streptomyces* sp., GETU-1 and GIG-1, could improve AMF root colonization and spore production under conditions of co-inoculation. Similarly, Fitter and Garbaye [81] reported that PGPMs, including actinobateria, increased the capacity of AMF to be colonized in root plants. The co-inoculation treatment involving *Streptomyces* sp. MCR9 and *Streptomyces* sp. MCR26 with *G. mosseae* increased the spore production of *G. mosseae* [77]. However, a significantly negative correlation in AMF root colonization and spore production was found in the co-inoculation treatment of *Amycolatopsis* sp. GLM-2 and AMF spores. Nevertheless, the mechanisms of actinobacteria that play a role in the stimulation or suppression of both AMF root colonization and spore production are not yet fully understood. One possible hypothesis has proposed that volatile and/or non-volatile compounds are produced by actinobacteria in the stimulation or suppression of AMF spore germination, mycorrhizal development, and spore production [82,83,84]. Carpenter-Boggs et al. [83] reported that volatile compounds produced by different species of *Streptomyces* could stimulate the germination of AMF spores and hyphae growth. Barea et al. [85] reported that PGPMs, including actinobacteria, are able to produce phytohormones (IAA and indole-like compounds) that not only enhance plant growth, but can also influence the establishment of AMF, as well as spore and hyphal growth. Additionally, the interactions between AMF and actinobacteria are influenced by different factors including the AMF species and actinobacterial strains, plant species, rhizospheres, and certain climate properties [83,84,86,87].

## 5. Conclusions

Presently, the search for sources of effective plant growth microorganisms that could be applied in sustainable agriculture has increased. In the present study, three actinobacteria associated with arbuscular mycorrhiza fungal (AMF) spores were isolated and identified. All the obtained actinobacterial strains were characterized as PGPMs and were capable of producing IAA, siderophores, endoglucanase, and ammonia by the in vitro assay. Moreover, they were found to be tolerant to drought, pH, salinity, and some agrochemicals. The obtained actinobacteria associated with AMF spores should be considered as a viable PGPM by promoting the growth of chili. Our findings indicate that the co-inoculation of *Streptomyces* sp. GETU-1 and AMF (*C*. *etunicatum*) spores enhanced the growth and yields of chili plants more effectively than in treatments involving a single strain inoculation and non-inoculation under greenhouse conditions. Furthermore, the co-inoculation of *Streptomyces* sp. GETU-1 and AMF also increased both AMF spore production and colonization in chili roots. Future studies will focus on the application of the co-inoculation treatment involving the obtained actinobacterial strains and AMF in terms of the growth and production capabilities of different plants and field experiment. This will be done to help researchers more fully understand the process of developing effective biofertilizers and bioenhancers that could replace the environmentally harmful chemicals and fertilizers currently being used in the field of agriculture. Clinical tests on toxicity will be required in these future studies to confirm the safety of these actinobacterial strains.

## Figures and Tables

**Table 1:** The treatment detail in this study.

Number	Treatment	Detail
1	Control	Control (uninoculated microorganisms)
2	AMF	Inoculation of *C. etunicatum* PBT03
3	(GETU-1)	Inoculation of *Streptomyces* sp. GETU-1
4	(GIG-1)	Inoculation of *Streptomyces* sp. GIG-1
5	(GLM-2)	Inoculation of *Amycolatopsis* sp. GLM-2
6	(AMF + GETU-1)	Inoculation of *C. etunicatum* PBT03 and *Streptomyces* sp. GETU-1
7	(AMF + GIG-1)	Inoculation of *C. etunicatum* PBT03 and *Streptomyces* sp. GIG-1
8	(AMF + GLM-2)	Inoculation of *C. etunicatum* PBT03 and *Amycolatopsis* sp. GLM-2

**Table 2:** Isolation source and characteristics of the obtained actinobacteria in this study.

Information	Actinobacterial Strain		
	GETU-1	GIG-1	GLM-2
Characteristic on the ISP-2 agar			
Aerial mycelium	Pale Greenish Yellow	Pale Greenish Yellow	Yellowish White
Substrate mycelium	Light Greenish Yellow	Strong Yellow	Vivid Yellow
Aerial mass color	Grayish Greenish Yellow	Grayish Olive	White
Soluble pigment	Absent	Absent	Absent
Spore chain morphology	Spirals	Spirals	Retinaculum-apertum
Diaminopimelic acid	LL–DAP	LL–DAP	*meso*–DAP
Classification	Streptomycete group	Streptomycete group	Non-streptomycete group

**Table 3:** Plant growth promotion properties of actinobacteria in this study.

Plant Growth Promotion Properties	*Streptomyces* sp. GETU-1	*Streptomyces* sp. GIG-1	*Amycolatopsis* sp. GLM-2
IAA production (µg/mL)	4.91 ± 0.43 ^b^	24.30 ± 4.16 ^a^	3.04 ± 0.22 ^b^
Siderophore production	+	+	+
Solubilization of phosphate mineral	–	–	–
Solubilization of potassium mineral	–	–	–
Endoglucanase production (EAI)	2.84 ± 0.10 ^b^	3.47 ± 0.20 ^a^	2.15 ± 0.40 ^c^
Chitinase production (EAI)	–	–	–
Ammonia production	+	+	+
Drought tolerance (a_w)_	0.957–0.998	0.957–0.998	0.897–0.998
pH tolerance	5–11	5–12	4–11
Salinity tolerance (% NaCl)	Up to 2.5%	Up to 2.5%	Up to 2.5%

EAI: Enzyme activity index; “+”: Positive result; “–”: Negative result. Results of IAA and enzyme production are means ± standard deviation and the same superscript letter in a row are not significantly different at the *p* < 0.05 by the DMRT.

## Data Availability

The DNA sequence data obtained from this study have been deposited in GenBank under accession numbers; MW897732, MW897733, and MW897734.
